# Meeting February 15th

**Published:** 1886-04

**Authors:** 


### CHICAGO MEDICAL SOCIETY.

*Stated Meeting, February 15th, 1886.* The President, C. T. PARKES, M.D., in the chair.

Dr. WILLIAM T. BELFIELD gave a brief outline of SEVEN CASES OF DIGITAL EXPLORATION OF THE BLADDER IN THE MALE, as follows:

CASE 1.—A patient, 58 years old, who had been treated for several years, by various physicians, for cystitis and prostatic enlargement; complete retention had occurred several times. A diagnosis of villous tumor of the prostate was made, the nature of the growth being predicated upon the semi-gelatinous state which the urine presented after standing for a few minutes—an almost pathognomonic symptom when present. Exploration revealed a small villous growth just to the left of the urethral orifice in the bladder; and embedded in its meshes were found two stones, as large as a pea and a bean respectively, whose existence had not been suspected. Patient made a rapid recovery, and was entirely free from his old complaint three months afterwards.

CASE 2.—A boy, 17 years old, had for two years suffered from frequent and painful urination, during which time considerable pus was constantly present in the urine. A probable diagnosis of tuberculosis of the urinary tract was made, and was confirmed some time afterward by the discovery of the characteristic bacilli in the urinary pus. At the urgent request of the parent the bladder was explored, with negative result. Some months afterwards nodular enlargement of the prostate and epididymis were developed. Patient's symptoms unimproved by the operation.

CASE 3.—A man, 63 years old, in the last stages of chronic cystitis, was admitted to the County Hospital. Had been treated for enlarged prostate. No enlargement of this gland could be recognized, but there was found, upon rectal examination, a collection of calculi occupying the exact site of the left seminal vesicle. Upon exploration of the bladder it was found that these calculi were contained, not in the vesicle, but in a diverticulum of the bladder, where they were completely encysted and covered with mucous membrane. This covering was torn and seventeen stones were removed. The patient's condition was much improved for two weeks after the operation, but at the end of six weeks he died.

CASE 4.—A man, 38 years old, suffering from all the symptoms of an extremely tight stricture of the deep urethra. No stricture could be found. Upon exploration of the bladder there was discovered a pedicled cyst attached to the upper orifice of the urethral opening, and completely occluding the urethra like a valve ; the tumor appeared to be as large as a small walnut. During an attempt at removal with forceps the cyst collapsed, about half an ounce of clear fluid escaping. The patient recovered rapidly, and has since remained quite free from urinary irregularities. This appears to be the first recorded instance of simple cyst in the male bladder.

CASE 5.—A patient, 38 years old, admitted to the hospital in a typhoid condition, suffering from cystopyelitis for which no cause was discovered. Exploration of the bladder showed nothing abnormal. The cystitis and pyelitis subsided completely. When patient was able to leave the hospital, it was discovered that he was suffering from an incipient myelitis.

CASE 6.—A man, 31 years old, admitted to the hospital with violent and fetid cystitis. It was found impossible to pass any instrument over a prostatic obstruction into the bladder ; from the rectum the prostate was found irregularly enlarged. Upon exploration of the bladder, a malignant tumor, involving the prostate and a large part of the bladder wall, was discovered. Complete anuria ensued, and death from uræmia on the fourth day. At the autopsy, hydronephrosis of the left kidney, caused by a pipe-stem calculus of the ureter, was discovered.

CASE 7.—A man, 59 years old, was admitted to the hospital for a chronic cystitis of fifteen years' standing, caused by prostatic enlargement. Perineal incision was made for temporary relief. Three weeks later a portion of the obstructing prostate was removed through the perineal wound by the galvano-cautery. Complete recovery. The patient, who had for two years been practically unable to urinate except through a catheter, now passed water freely without assistance ; the cystitis subsided. Nine months afterwards, acute uræmia and death. At the autopsy, small cirrhotic kidneys were found. This is believed to be the first case recorded in which a considerable portion of the hypertrophied prostate has been deliberately and intentionally removed.

On concluding his paper DR. BELFIELD spoke of a case which came into the hospital in a state of extreme exhaustion, evidencing violent cystitis. Urine containing pus was passed every fifteen minutes with extreme pain. It was impossible to get an instrument into the bladder more than half an inch. The man was half unconscious and could give no history, but by rectal examination it was discovered that the prostate was very much enlarged in the left lobe, the right lobe appearing normal in consistency and size. Dr. Belfield thought this almost a proof that in a man of this age, 31, the seeds of tuberculous disease were present. The bladder was opened and the finger inserted in the usual way, discovering a malignant growth covering an inch and a half of the left wall of the bladder. After the operation the patient passed half an ounce of urine, but died in three days from uræmia. The bladder and kidneys were secured and found to be the seat of extensive hyperinosis, caused by a calculus. Another case was that of an emaciated French Canadian who had had trouble for eighteen years, and had depended entirely for urination upon the catheter for two years. The usual perineal incision was made, and a portion of the prostate removed. The operation was successful and the man was able to do without the catheter, although Dr. Belfield advised its use once a day. In June he had a slight attack of cystitis, due to the fact that he had persisted in ignoring the advice in regard to the use of the catheter. About the 10th of September he was taken with uræmic convulsions, and died on the 18th. The last few days the urine became cloudy again. The bladder and kidneys were secured, and a channel found in the prostate large enough to admit the forefinger. Dr. Belfield thought it would have been better to have cut down from above in the hope of relieving the prostatic tumor.

PROFESSOR E. ANDREWS suggested to the members that this subject was more important than was formerly supposed. The former examinations made in these cases were very imperfect, and though the finger used occasionally to be put in the bladder, it was not dreamed that the whole circumference of it could be reached. He thought it should be impressed upon the members of the profession that the bladder can and ought to be explored.

THE PRESIDENT thought this procedure for diseases of the bladder so new that it might be called novel, and it seemed singular that at so late a day the bladder should be explored in this way, when surgeons have so long known that the bladder could be opened in many ways without injury to the patient. He thought the profession greatly indebted to Dr. Belfield for his remarks.

DR. W. L. AXFORD thought an incision in the perineum might facilitate a cure in cases of fistula, which at present are very difficult to cure, but did not know that it had ever been tried. We do know that renal fistulæ are very difficult to cure, probably because the urine is continually running over them. The procedure is practically harmless and may effect a cure.

DR. W. T. BELFIELD said in conclusion that this procedure of inserting a drainage tube through the perineal wound for curing fistula of the urethra is mentioned in Harris's book, and seems to work with entire success. There was one point upon which he congratulated himself, viz., the discovery of cyst of the bladder. So far as he knew, nothing of the kind had been recorded in either the pathological or clinical literature of the bladder. The misery that it can cause is illustrated in the case under discussion, and from its nature it offers every hope of a cure.

DR. W. L. AXFORD read a paper on

NOTES ON GENITO-URINARY SURGERY, in which he said that he believed gleet is due to a stricture of the urethra, and can be cured by curing the stricture. He believed that dilatation cured a stricture by exciting inflammation at the seat of the stricture, and thus causing the absorption of the exudate. In the treatment of strictures he believed that perineal section is best in strictures which are deep-seated, narrow, or unyielding, or complicated by fistula or urinary infiltration; internal urethrotomy in all strictures of the meatus or fossa navicularis, for they will not stretch. For other strictures he would advise, as the case demanded, dilatation, divulsion, or internal urethrotomy. If the stricture is resilient, divulsion or internal urethrotomy, preferably the latter. He believed the dull pain in the back, and at times in the head, during the later stages of gonorrhœa, to be due to urethral reflexes. Cure the gonorrhœa or stricture and these disappear.

He cited cases in which severe cephalalgia and skin diseases had been cured by curing strictures by internal urethrotomy. Also, cases in which dysuria and vesical discomfort had been allayed by slitting the meatus. In female urinary troubles, after excluding uterine disease, he had given relief by digital dilatation of the urethra.

PROFESSOR E. ANDREWS referred to the point whether there is any danger or not in urethrotomy. European statistics show a considerable amount of danger in operations for stricture. He did not know the reason for mortality in Europe, for he thought it the general experience in Chicago that men don't die of these operations. Long ago he had left off keeping count of the cases he had operated on, and could not recall an instance in his own practice or that of any one else in this city in which the patient died in consequence of the operation for stricture. As to the question of divulsion or cutting, he had performed the two operations indiscriminately, sometimes both on the same patient. He thought divulsion and cutting produce precisely the same result. Some strictures will stretch out under the divulsor and go back again, and cutting is better for these cases, but neither appears to be dangerous. He had found divulsors from New York instrument-makers repeatedly breaking down under strong strictures. Otis's instrument he thought exceedingly strong, but even that had failed in one instance. He expressed the belief that divulsion, thoroughly earried out, is as safe as cutting.

PROFESSOR F. E. WAXHAM referred to a unique case coming under his care of stricture in a child aged four years. He was convinced, however, that stricture is very rare among children. This was the only case that had come under his observation. Otis alludes to two causes—masturbation and gonorrhœa. In this case he found no history of gonorrhœa, and the mother denied the practice of masturbation. The child was put under the influence of ether; the stricture was so closed that he could only enter the bladder by means of a very fine bougie; this was followed by a larger one, this by a small catheter, and this by a steel sound, and the child was then able to pass the urine without difficulty. Before dilatation the child would undergo the greatest suffering every time he passed water, would scream and strain for several minutes, and oftentimes strain so severely as to cause evacuation of the bowels. After the firs dilatation the child did nicely for a week and then was as bad as ever; the second dilatation was three weeks ago, and there has been no further trouble.

DR. A. B. STRONG asked if the cure was permanent after cutting unless constantly followed up with the catheter. He wished to know if there is anything better than simple dilatation. Cutting and divulsion are more rapid, but he thought gradual dilatation offered as good and permanent results.

DR. W. T. BELFIELD suggested two points in Dr. Axford's paper that required notice, the value and the safety of cutting. He thought it absolutely imperative that we limit our cutting operations strictly to the pendulous urethra. This was not merely a theoretical and anatomical consideration, but a practical one. He thought Dr. Axford in speaking of the three methods, stretching, divulsion and cutting, omitted one of the most importance, viz., the use of a constant electric current. Although this had not received a great deal of attention from the profession, he thought anybody who tried it would never give it up. It is said to produce the most permanent results, and is convenient and easy. He had tried it in his own practice, once in the hospital where he had the privilege of cutting, but tried the battery out of curiosity and found it worked nicely, and again in private practice where it was extremely important to the patient that no perineal section should be done. As to stretching the female urethra with the fingers, he thought it dangerous. When house surgeon in the County Hospital he had seen a woman die of peritonitis when that was done. He thought the best instrument for this purpose to be Stein's dilator, which has a continous dilatation.

DR. W. L. AXFORD said, in conclusion, that dilatation always had to be followed out by the occasional passage of the sound for an indefinite period of time; all the patient's life. If the stricture is divided clear through, Dr. Otis claims that perfect cure will be made and no after treatment will be necessary. Dr. Axford thought that Dr. Otis, although an extremist on this subject, is about right. As to cutting in the membranous urethra, he did not think any one would do that. He never had experience with the galvanic current, had heard it recommended and also discountenanced. In dilatation of the female urethra he had always used his fingers and succeeded first rate. Would always prefer to use his own hand, as no instrument equals the human hand.

DR. H. T. BYFORD read a paper on the

PRODUCTION AND PREVENTION OF LACERATION OF THE PERINEUM, with description of an unrecognized form. Illustrated. For the purposes of illustration the perineum was divided into two parts, the vulval or external, and the vaginal or internal. The former lies external to the muscles and the latter includes all the rest. When the perineum is slightly stretched by the head two rings may be felt by hooking the finger over the fourchette; an external ring marking the edge of the vulva and an internal ring marking the edge of the muscles and fascia about the vaginal orifice. Upon the positions and relations of these rings depends the safety of the perineum. When the occiput engages in them before the forehead passes the coccyx, the fourchette and muscular edge are pressed downwards and the perineum but little bulged and not at all endangered. When the occiput does not engage in the rings before the forehead leaves the coccyx, the fourchette is pushed upward before it, the perineum stretched from three to four inches antero-posteriorly, while part of the propelling force and nearly all of the directing force are lost before the head passes the rings and is born. The head in such cases has a greater distance to travel before delivery, involving loss of time and strength.

The author described an obscure but not infrequent form of rupture in which separate muscular fibres and portions of the fascia give way all through the perineum, or through a region of it, without involving skin or mucous membrane. Frequently there were not enough unruptured fibres left of the tissues to insure contraction and involution, and thus prevent displacements of the uterus. A persistent flabbiness is the chief diagnostic sign. To prevent rupture Dr. Byford advised: 1. To gain time for dilatation without injury to the deeper tissues by favoring a slow advance of the head over the floor of the pelvis. 2. In order to secure sufficient dilatation of the vulval and vaginal rings to make the occiput ride over them instead of hooking under them by keeping the membranous pouch intact, or in its absence early resort to artificial dilatation rather than wait for the fourchette to be thus hooked up over a bulging perineum, and then resort to the ordinary methods.

DR. G. H. RANDALL thought that by directing the head many perineums might be prevented from rupturing. We sometimes fail to direct the head and manipulate the perineum as each case requires because the woman is not in the proper position. Formerly he had delivered most of his cases in the dorsal position, but not being satisfied with the way he was able to manipulate the perineum and head, he had tried the lateral position, and it seemed a great improvement. He could now support the perineum by directing the head with a great deal more ease and effect than ever before, and it seemed to him that the lateral position is preferable to the dorsal.

Owing to the lateness of the hour PROFESSOR R. G. BOGUE deferred reading his paper on "A Case of Oöphorectomy and of Nephrectomy," but exhibited specimens, and said: "The specimen that I have here is that of a remarkable cystic ovary in which both ovaries, with a large portion of the Fallopian tubes, were removed. I have but one to present, though the whole surface of the ovary, or ovaries, was studded with small cystic tumors. The case was one of long-standing invalidism, and attended with a great deal of suffering. Finally the question of removal of the ovaries came up, and it was decided to remove them. They were removed and the patient recovered.

The other specimen that I have is a wax model of a calculus which was found in the pelvis of a kidney during an operation for removal of fibro-nephroid kidney. On opening the loin, making the posterior operation and cutting of the kidney, it was found to be immensely distended and filled with pus, and in the pelvis of the kidney was quite a large calculus. The calculus was claimed by the family and given them, a wax cast of it being retained. The patient from whom this was removed did not recover."

Exhibition of pathological specimens from Batty's operation, and specimens of ovarian tumor with twisted pedicle, by PROFESSOR C. T. PARKES, who said:

"The specimens which I have to present to you on this plate are two ovaries and forty gall stones. The two ovaries were removed from a lady who had suffered for over ten years from a great deal of trouble in the pelvis. They are noticeable in that the left one is very small and the right one very large.

In this box are forty-three gall-stones which I removed from a gall-bladder yesterday.

In reference to the oöphorectomy, I desire to call attention to the knot used in securing the pedicle and which has been rendered famous by Mr. Tait; he calls it the Staffordshire knot. Its use got me into trouble. It does not secure the pedicle by merely tying the knot. It must be drawn sufficiently tight to cut off circulation in the pedicle before the final knot is secured, otherwise the pedicle is very feebly constricted. I used it in this case in securing the left ovary, and passed on to the removal of the right ovary, during which operation I noticed that a good deal of bleeding was going on. Having removed the right ovary, I then looked for the point of hæmorrhage and found a spurting artery in the unsecured left pedicle. I was astonished, because I thought I had tied the knot very tightly. It is necessary that the constriction should be made by the string, and the circulation must be entirely cut off before any attempt at tying is made. The other specimen is a large tumor, the weight of which was estimated at twenty-three pounds. Eight days before the operation the patient, having been in a previous condition of apparent good health, was suddenly taken sick, temperature 102° F., pulse very fast and feeble, abdomen tender from peritonitis, urine suppressed. I diagnosed a twisted pedicle, and advised immediate operation. The operation was done as soon as possible, and although she was in a very feeble condition at the time, the operation apparently made very little impression upon her. As soon as the abdominal incision was made a black tumor presented, and instead of the cyst portion being uppermost, as is almost invariably found, the solid portion was in front. When the trocar was pulled out there was scarcely any hæmorrhage from the opening made by it; usually there is considerable hæmorrhage, especially when introduced into a solid tumor. When it was removed from the abdomen there was found to be a turn and a half in the pedicle. The temperature fell after the operation and did not again come up to one hundred. On the seventh day all the stitches were removed, and the patient is practically well to-day. These tumors sometimes have pedicles twisted completely off, so that they are in a sloughing condition; in other cases the twisting goes on so slowly that the pedicle is finally destroyed entirely. Spencer Wells says that he has found tumors with no pedicles. The three points that seem to me to indicate this diagnosis are the rapid occurrence of distension, the commencement of peritonitis, and the suppression of urine."

The President said, in answer to a question, that because a patient has symptoms of gall-stones and the evidences are pretty positive of their presence, no one must imagine that thereby he is going to have an easy time of the operation. The case from which these gall-stones were removed was a very difficult one for operation. The patient was a woman of considerable adipose tissue on the surface of the body, and it was difficult to expose the gall-bladder at all. The liver, instead of being distended and projected below the ribs, was contracted and high above them. It was difficult to find the gall-bladder at first, and it was found to be about the size of a finger, elongated and lying in its natural position and adherent to the liver. The fundus was contracted and hardened or thickened so that the finger had to be passed well down along its surface before any evidence of the stones could be found at all; it was separated from its attachments to the under surface of the liver, and then by carrying a thread through its fundus it was lifted to the top part of the incision and the finger introduced into the cavity of the gall-bladder and the stones removed. Of these stones the largest was found in the gall-duct. It was forced from the gall-duct into the gall-bladder.

DR. H. P. MERRIMAN asked the President if any cause was known for the twisting of the pedicle in ovarian tumor.

THE PRESIDENT said that he made very close inquiry on that point; the night previous to the occurrence of the trouble the patient felt something move in the abdomen from the left to the right side. She was lying in bed at the time. In reply to Dr. Strong the President said he did not think that rupture of the cyst would not be followed by an increase in the size of the abdomen, nor by any change in the function of the kidney.

DR. W. FRANKLIN COLEMAN read a paper on

A REPORT OF THREE CASES OF OSSIFICATION OF THE CHOROID, AND THE REPORT OF ONE CASE OF OSSIFICATION OF THE LENS, WITH SPECIMENS.

In cases one, two, three and four, the eye had been lost five, twelve, fourteen and thirty-four years respectively before medical advice was sought, on account of sympathetic trouble in the fellow eye. In cases one, two and four, the lost eye had occasionally been painful. In cases one, two, three and four, sympathetic disease did not occur after the loss of the eye until five, twelve, eleven and eighteen years respectively. The sympathetic disease excited in case one was serous kerato-iritis; case two, cyclitis; case three, irido-cyclitis and cataract; case four, optic neuritis and mild iritis. Dr. Coleman advised the immediate enucleation of a bony eye on the same ground that the enucleation of an eye lost from injury in the ciliary region would be advised. Neither the ossification nor the injury is, in itself, the immediate cause of the sympathetic disease, but either may be the indirect cause by exciting an iride-cyclitis.

DR. R. TILLEY said the term ossification of the lens might lead to a misunderstanding that there is actually ever an ossification of the lens. Becker, of Heidelberg, claims that ossification of the lens cannot and does not take place, and that there is no case of it on record. He says that in the case of a ruptured capsule, a membrane may be developed from the ciliary region, and from the developing blood-vessels an ossification may take place in the region of the lens, but that it should not be called an ossification of the lens. He gives an interesting case showing how readily a mistake may be made and ossification supposed: A boy was struck in the eye with a hay fork, and came under his care about ten hours after the accident. Forty-three hours after the accident the eye was enucleated, and he thought from a macroscopic examination that the lens was intact, but on microscopic examination, what he supposed to be the lens was found to be an extravasation of blood, and the lens had escaped. He remarked that it is easy to see that an ossification might have occurred, under these circumstances, and the same been taken for the lens.

DR. COLEMAN said, in conclusion, that he was well aware that literally and truly the lens fibres do not become ossified, but wherever connective tissue exists ossification may occur, and connective tissue may occupy the site of the lens. Dr. Voorhies cites a case in his own practice of an ossific mass occupying the normal position of the lens which an expert microscopist, upon examination, pronounced ossification of the lens. Dr. Knapp does not say he denies the possibility of such ossification.

